# Effects of acupuncture on the outcome of tinnitus: An overview of systematic reviews

**DOI:** 10.3389/fneur.2022.1061431

**Published:** 2022-11-18

**Authors:** Xianpeng Xu, Hui Xie, Zifeng Liu, Tao Guo, Ying Zhang

**Affiliations:** Department of Otorhinolaryngology, Hospital of Chengdu University of Traditional Chinese Medicine, Chengdu, China

**Keywords:** tinnitus, systematic reviews, AMSTAR-2, GRADE, PRISMA, acupuncture

## Abstract

**Background:**

To systematically summarize the evidence for the efficacy of acupuncture in tinnitus treatment, we assessed the methodological quality, reporting quality, and evidence quality of systematic reviews/meta-analyses (SRs/MAs) of acupuncture in the treatment of tinnitus.

**Methods:**

From inception to March 2022, we conducted a detailed and comprehensive search of eight electronic databases in Chinese and English. The Assessing the Methodological Quality of Systematic Reviews 2 (AMSTAR-2), the Preferred Reporting Items for Systematic Reviews and Meta-Analyses (PRISMA) checklist, and the Grading of Recommendations Assessment, Development, and Evaluation (GRADE) were used to assess methodological quality, reporting quality and evidence quality for inclusion in SRs/MAs, respectively.

**Results:**

Fourteen published SRs/MAs met the inclusion criteria and were included in the study. Eleven studies reported that acupuncture was effective in treating tinnitus, and three studies reported that no firm conclusions could be drawn about the effectiveness of acupuncture in treating tinnitus. The results of the AMSTAR-2 assessment showed that the methodological quality of the included studies was relatively low in general, with one being moderate quality and the rest being very low quality. The PRISMA checklist evaluation results showed that no studies fully report checklists, with protocol registration and search strategies being the main reporting weaknesses. The GRADE assessment showed that no results were high-quality evidence, 17 results were moderate-quality evidence, 25 results were low-quality evidence, and 12 results were very low-quality evidence.

**Conclusion:**

Acupuncture seems to be a positive and effective treatment for tinnitus. However, the methodological quality and quality of evidence for SRs/MAs in the included studies were generally low, and this result must be viewed with caution. Therefore, more high-quality, large-scale, multi-center randomized controlled trials are needed in the future to verify the effectiveness of acupuncture in the treatment of tinnitus.

## Introduction

Tinnitus is the perception of sound in the absence of external acoustic stimulation (1). The sensation of the absence of external sound sources is a basic nature, and patients usually describe this sound as hissing, sizzling, and ringing, but also some complex sounds, such as music and voice (2). According to the National Health Interview Series, approximately 10% of respondents across the United States said they had experienced tinnitus in the past 12 months, which caused some difficulty in their lives (3). A systematic review and analysis of the prevalence and incidence of tinnitus in the world showed that, based on available data, more than 740 million people worldwide suffer from tinnitus in varying degrees, and more than 120 million people suffer from severe tinnitus (4). Currently, the pathological mechanism of tinnitus has not been elucidated, which may be related to the changes in neuronal activity in the central nervous system (5); moreover, the medical community has not yet found a cure for primary tinnitus, and no medications have been proven to alleviate tinnitus (6). Nevertheless, many therapies are still widely used in the treatment and research of tinnitus, including auditory therapy, cognitive behavioral therapy, transcranial magnetic stimulation, dietary supplement therapy, and acupuncture (6).

Acupuncture, as one of the indispensable and essential components of complementary and alternative medicine, has been widely used to treat many conventional and complicated diseases, including various functional and painful conditions (7). Currently, acupuncture has been shown to affect cochlear blood flow and cochlear nucleus, which may be one of the reasons acupuncture works in the treatment of tinnitus (8). Acupuncture, as physical therapy, is more likely to have an impact on patients due to its operation mode of deep contact with patients, and the superposition of clinical and situational factors has a profound and complex impact on patients (9). From the personal and psychological level, the treatment behavior of acupuncturists conveys a positive belief to patients that they are being cured, which is similar to the placebo effect of acupuncture; from the perspective of neurobiology, the acupuncture placebo effect may be through the release of certain neurotransmitters, on the neurochemical levels interact with brain control system (10).

However, it must be acknowledged that we cannot yet draw firm conclusions due to the lack of sufficient well-designed studies. Systematic reviews (SRs)/meta-analyses (MAs) are critical pieces of evidence for the development of clinical practice guidelines, and whether their conclusions are meaningful depends on the quality of the included studies (11). According to the SRs/Mas, we retrieved for the effectiveness of acupuncture in the treatment of tinnitus, we found that the conclusions of these studies are not entirely consistent; to address these controversial results and to further assess the quality of the evidence, we provide an overview of the SRs/MAs of acupuncture for tinnitus.

## Materials and methods

The overview methodology followed the Cochrane Handbook and the Preferred Reporting Items for Systematic Reviews and Meta-Analyses (PRISMA) (12).

### Criteria for considering reviews

#### Type of study

The study included SRs/MAs meeting the following criteria: (1) The studies included in SRs/MAs were limited to clinical randomized controlled trials (RCTs) or clinical controlled trials; (2) The languages for SRs/MAs were limited to English and Chinese. The exclusion criteria were as follows: (1) Not related to acupuncture, tinnitus, or SRs/MAs; (2) Letters, case reports, posters, graduate dissertations, and duplicate studies.

#### Types of participants

Study participants with a precise diagnosis were patients with primary tinnitus. Primary tinnitus definition: Tinnitus that is idiopathic opathica and may or may not be associated with sensorineural hearing loss (6). Secondary tinnitus, such as simple cerumen impaction of the external auditory canal, otosclerosis, eustachian tube dysfunction, vascular tumor, vestibular schwannoma, vascular anomalies, myoclonus, and intracranial hypertension, were excluded.

#### Types of intervention

The intervention in the treatment group was acupuncture therapy (such as acupuncture, electroacupuncture, body acupuncture, scalp acupuncture, warm acupuncture, and ear acupuncture) or acupuncture combined with other treatments. The control group could be medication therapy, traditional Chinese medicine therapy, cognitive behavioral therapy, auricular therapy, dietary therapy, and sham acupuncture or placebo acupuncture. However, the efficacy comparison of different acupuncture methods should be excluded.

#### Types of outcomes

The outcome measures of SRs/MAs included in this study had at least one of the following options: the clinical efficacy rate, improvement in quality of life, as assessed using a visual analog scale (VAS), Tinnitus handicap inventory (THI), adverse events (AEs), Tinnitus severity index (TSI), changes in the auditory threshold, improvement in annoyance, and awareness of tinnitus.

### Database and search strategy

From inception to March 2022, four Chinese electronic databases (China National Knowledge Infrastructure, Wanfang Database, Chongqing VIP, and Chinese Biomedical Literature Database) and four English electronic databases (PubMed, Cochrane Library, EMBASE, Web of Science) were systematically searched. Key phrases included “tinnitus,” “acupuncture,” “systematic review,” or “meta-analysis.” We conducted an updated search in September 2022 in order to prevent missing the most recent studies and provide the most comprehensive evidence. Table 1 represents the search strategy for PubMed. More search strategies are available in Appendix 1.

**Table 1:** Search strategy for Pubmed.

**Query**	**Search term**
#1	Tinnitus [Mesh]
#2	Tinnitus[Title/Abstract] OR Tinnit*[Title/Abstract] OR Ear and (Ring* or Buzz* or Roar* or Click* or Puls*)[Title/Abstract] OR Somatic Tinnitus [Title/Abstract] OR Idiopathic Tinnitus [Title/Abstract] OR Chronic Tinnitus [Title/Abstract] OR Acute Tinnitus [Title/Abstract] OR Pulsatile Tinnitus[Title/Abstract] OR Subjective Tinnitus [Title/Abstract]
#3	#1 OR #2
#4	Acupuncture [Mesh]
#5	Acupuncture[title/abstract] OR Dry Needling[title/abstract] OR Acupotomy[title/abstract] OR Acupotomies[title/abstract] OR Electroacupuncture[title/abstract] OR Body Acupuncture[title/abstract] OR Ear Acupuncture [title/abstract] OR Scalp Acupuncture [title/abstract] OR Warming Needle [title/abstract]
#6	#4 OR #5
#7	Meta-Analysis as Topic [mesh]
#8	Systematic Review* [Title/Abstract] OR Cochrane Review* [Title/Abstract] OR Meta-analysis [Title/Abstract] OR Meta-analyses
#9	#7 OR #8
#10	#3 AND #6 AND #9

### Data collection and extraction

The title and abstract of the articles were read by two independent reviewers (XP-X and ZF-L) and validated, screened, and extracted according to the inclusion criteria. Any disagreements were reached through discussion and negotiation or resolved through consensus and consultation with an experienced, authoritative third reviewer (H-X). The included studies extracted detailed features: author(s), year, publication language, number of included studies, sample size, treatment intervention, control intervention, quality assessment tool, relative effect (95% CI), P value and principal conclusions.

We extracted the studies included in each included review, using an Excel spreadsheet, to explore overlap in the literature and assess overlap. Graphical Representation of Overlap for OVErviews (GROOVE) (13) is an easy-to-use tool, where the matrices of evidence and the calculation of the corrected covered area (CCA) are probably one of the most exhaustive methods of measuring overlap. Using statistics, it calculates the coverage area CCA and provides an interpretation of the overall overlap assessment, which is slight if the CCA is < 5%, moderate if it is ≥5% and < 10%, high if it is ≥10% and < 15%, and very high if CCA is ≥15% (13).

### Quality assessment

Two independent reviewers (XP-X and ZF-L) used the Assessing the Methodological Quality of Systematic Reviews 2 (AMSTAR-2) (14), the Preferred Reporting Items for Systematic Reviews and Meta-Analyses (PRISMA) (12) checklist, and the Grading of Recommendations Assessment, Development, and Evaluation (GRADE) (15), to assess the methodological, reporting, and evidence quality of the included studies, respectively. Any disagreements were reached through discussion and negotiation or resolved through consensus and consultation with an experienced, authoritative third reviewer (H-X).

The AMSTAR-2 is an effective tool to evaluate the methodological quality of SRs/MAs, mainly used to critically assess the systematic review of randomized controlled clinical trials. The AMSTAR-2 includes 16 projects, each of which is a standardized problem, and the results are rated as “yes,” “partially yes” and “no”.

The PRISMA checklist provides reporting specifications for SRs/MAs and references for the standardized writing and reporting of SRs/MAs. Standardized reporting can reduce bias between research and published results and increase article transparency. The PRISMA checklist includes 27 projects, each of which is a standardized problem, and the results are rated as “yes,” “partially yes” and “no”.

The GRADE system is an internationally unified literature evaluation system for grading the evidence quality and the recommendations' strengths. The GRADE system divides the factors that may affect the quality of evidence from RCTs into five downgrading factors (risk of bias, inconsistency, indirectness, imprecision, publication bias) and three upgrading factors (a large effect, plausible confounding would change the effect, and dose–response gradient). The GRADE system details the factors that affect the quality of evidence and gives a standard for the level of evidence: high, moderate, low, and very low.

## Results

### Results of literature search and selection

**Figure 1:**
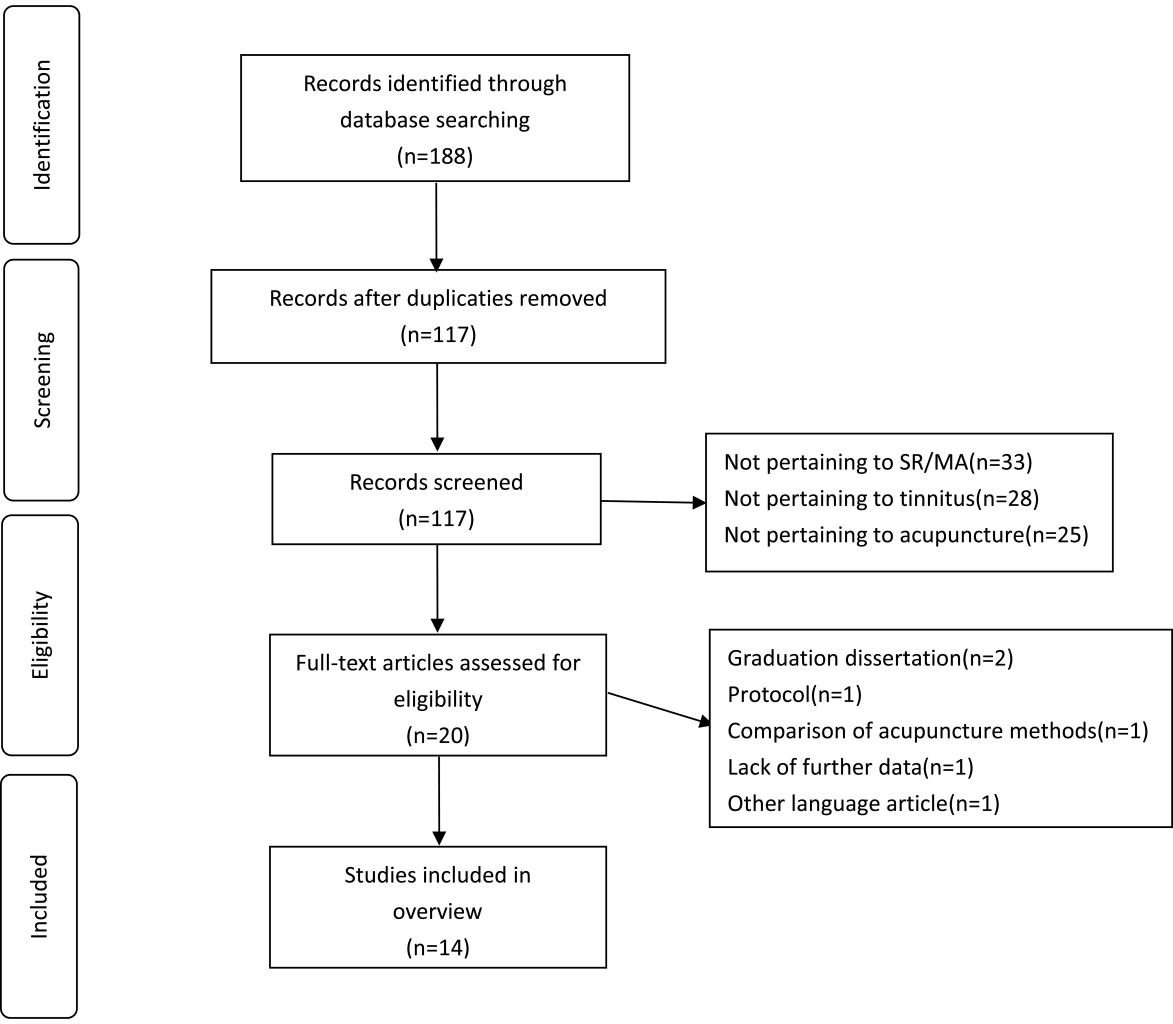
Flow diagram of the literature selection.

According to the established search strategy, we conducted a preliminary search and searched 188 articles. A total of 71 duplicate articles were excluded, and after reading the title and abstract, 33 articles were excluded not related to SRs/MAs, 28 articles not related to tinnitus, and 25 articles not related to acupuncture were excluded. Six articles that were read in full were excluded for the following reasons: graduation dissertation, protocol, comparison of acupuncture methods, lack of further data, and another language article. A total of 14 systematic reviews (16–29) finally met the requirements and were included. The process and results of the literature screening are shown in Figure 1.

### Characteristics of included studies

The 14 studies were published over a significant period, spanning from 2000 to 2020. Of which nine were published in Chinese and five were published in English. The number of RCTs included in SRs/MAs was significant, ranging from 5 to 25 eligible trials and 186 to 2,654 sample sizes. There were also slight differences in the intervention measures in the treatment group, among which 12 articles were mainly acupuncture in the treatment group, and two articles were mainly electroacupuncture; the intervention measures in the control group were similar, primarily using sham acupuncture and conventional treatment. To assess the quality of the original articles, the Jadad scale was used for six articles and the Cochrane risk of bias criterion was used for eight articles. The main characteristics of the study are detailed in Table 2.

**Table 2:** Main characteristics of the studies.

**Author(s), year**	**Language**	**Trials (subjects)**	**Treatment intervention**	**Control intervention**	**Quality assessment**	**Relative effect (95% CI)**	***P*-value**	**Main conclusions**
Gong et al. (16)	Chinese	7(655)	AT,AT+CPM	CM, CPM	Cochrane criteria	/	/	Acupuncture is effective in treating tinnitus with few adverse reactions
Zhang et al. (17)	Chinese	17(1138)	AT	SAT,CT	Jadad	RR 1.25 (1.12, 1.32)	< 0.01	The effect of acupuncture therapy on tinnitus is better than that of the drug control group
Song et al. (18)	Chinese	9(1174)	AT	CT	Cochrane criteria	OR 3.26(2.09, 5.08)	< 0.0001	The total effective rate of acupuncture is better than that of conventional drug therapy. However, the support and argument of a large sample is still needed
Ji et al. (19)	Chinese	17(1637)	AT,AT+CT	CT	Jadad	/	/	Acupuncture is beneficial in treating tinnitus, but the evidence remains insufficient
Meng et al. (20)	Chinese	25(2654)	AT,AT+CT	AT,CT	Jadad	OR 2.98 (1.33, 3.97)	< 0.0001	Acupuncture is effective in treating tinnitus, and the total effective rate and cure rate of the treatment are higher than drug treatment
Nie et al. (21)	Chinese	6(528)	AT+CT	CT	Cochrane criteria	OR 2.80 (1.80, 4.35)	< 0.01	For tinnitus patients, acupuncture therapy has relatively good clinical efficacy
Ma et al. (22)	Chinese	15(1082)	AT,AT+CT	SAT,CT	Jadad	0R 3.68 (2.62, 5.26)	< 0.01	For neurogenic tinnitus, acupuncture or acupuncture combined with other therapies is superior to medication alone or non-acupuncture
Fang et al. (23)	Chinese	14(1202)	EAT,EAT+CT	AT,CT	Cochrane criteria	RR 1.21 (1.15, 1.27)	< 0.0001	Electroacupuncture is an effective therapy for tinnitus without obvious adverse reactions
Xie et al. (24)	Chinese	15(1153)	AT,AT+CT	CT	Jadad	/	/	Acupuncture treatment of tinnitus may alleviate the symptoms of the patient's ears
Liu et al. (25)	English	18(1086)	AT,AT+CT	SAT,CT	Cochrane criteria	RR 2.49 (1.06, 5.84)	0.04	Acupuncture may have subjective benefits for some patients with tinnitus
He et al. (26)	English	5(322)	EAT,EAT+CPM	AT,CT	Cochrane criteria	/	/	Electroacupuncture is effective for tinnitus in the short term; however, there are insufficient data to provide any support for long-term efficacy and safety
Huang et al. (27)	English	8(504)	AT,AT+CT	SAT,CM	Cochrane criteria	MD−1.81 (−3.69, 0.07)	0.06	For tinnitus, acupuncture had no significant effect on the primary outcome of the VAS score. However, the acupuncture group had a positive effect on secondary outcomes (THI and TSI scores)
Kim et al. (28)	English	9(432)	AT	SAT,CT	Cochrane criteria	/	/	Evidence of effectiveness of acupuncture for tinnitus is not convincing
Park et al. (29)	English	6(186)	AT,EAT	SAT,CT	Jadad	/	/	Acupuncture has not been shown to be effective in treating tinnitus, according to evidence from rigorous randomized controlled trials

AT, acupuncture; SAT, sham acupuncture; EAT, electroacupuncture; CT, CT, conventional therapy; CPM, Chinese patent medicine. OR, odds ratio; RR, relative risk/risk ratio; MD, mean difference.

### Methodological quality

The AMSTAR-2 was used to assess the methodological quality of included studies. Of the 14 included studies, only one was moderate, and the rest were very low. Only one article (27) met the criteria for item 2 affecting literature quality, which was conducted according to the reporting guidelines and standards set out in the PRISMA checklist and was registered in the PROSPERO database. None of the included studies met the key item 7 “yes” criteria. The evaluation details of other projects are shown in Table 3.

**Table 3:** Results of the AMSTAR-2 assessment.

**Included studies**	**AMSTAR-2**																**Overall quality**
	**Q1**	**Q2**	**Q3**	**Q4**	**Q5**	**Q6**	**Q7**	**Q8**	**Q9**	**Q10**	**Q11**	**Q12**	**Q13**	**Q14**	**Q15**	**Q16**	
Gong et al. (16)	Y	PY	Y	PY	N	N	N	Y	Y	Y	Y	Y	Y	Y	Y	N	CL
Zhang et al. (17)	Y	PY	Y	PY	Y	Y	N	Y	Y	Y	Y	Y	Y	Y	Y	Y	CL
Song et al. (18)	Y	PY	Y	PY	Y	Y	N	Y	Y	Y	Y	Y	N	Y	N	N	CL
Ji et al. (19)	Y	PY	Y	PY	Y	Y	N	N	Y	N	Y	Y	N	Y	N	N	CL
Meng et al. (20)	Y	PY	Y	PY	N	N	N	Y	Y	N	Y	Y	Y	Y	Y	N	CL
Nie et al. (21)	Y	PY	Y	PY	N	Y	N	Y	Y	N	Y	Y	Y	Y	Y	Y	CL
Ma et al. (22)	Y	PY	Y	PY	Y	Y	N	Y	Y	Y	Y	Y	Y	Y	Y	Y	CL
Fang et al. (23)	Y	PY	Y	PY	Y	Y	N	Y	Y	Y	Y	Y	N	Y	N	N	CL
Xie et al. (24)	Y	PY	Y	PY	Y	Y	N	N	Y	Y	Y	N	Y	Y	Y	Y	CL
Liu et al. (25)	Y	PY	Y	PY	Y	Y	N	Y	Y	Y	Y	Y	Y	Y	Y	Y	CL
He et al. (26)	Y	PY	Y	Y	Y	Y	N	N	Y	Y	N	Y	Y	Y	Y	Y	CL
Huang et al. (27)	Y	Y	Y	PY	Y	Y	N	Y	Y	Y	Y	Y	Y	Y	Y	Y	M
Kim et al. (28)	Y	PY	Y	Y	Y	Y	N	Y	Y	Y	N	Y	Y	Y	Y	Y	CL
Park et al. (29)	Y	PY	Y	PY	Y	Y	N	Y	N	Y	N	N	Y	Y	N	Y	CL

Y, yes; PY, partial yes; N, no; CL, critically low; L, low; M, moderate.

### Reporting quality

For the 27 projects on the PRISMA checklist, none of the included studies met all projects; however, most projects were also fully reported, with more than half of them achieving a 100% completion rate. Regarding specific details, Q5 (protocol and registration) was the most difficult to succeed in; only one study completed the review protocol and provided registration information and number. The Q8 (search) completion rate was low at 21.4%. Most studies only give a simple search strategy rather than a detailed complete search strategy that can be easily reproduced. Other projects whose completion rate did not reach 100% include Q9, Q15, Q16, Q19, Q22, Q23, Q25, and Q27. More details are presented in Table 4.

**Table 4:** Results of the PRISMA checklist.

**Section /topic**	**Items**	**Gong et al. (16)**	**Zhang et al. (17)**	**Song et al. (18)**	**Ji et al. (19)**	**Meng et al. (20)**	**Nie et al. (21)**	**Ma et al. (22)**	**Fang et al. (23)**	**Xie et al. (24)**	**Liu et al. (25)**	**He et al. (26)**	**Huang et al. (27)**	**Kim et al. (28)**	**Park et al. (29)**	**Compliance (%)**
Title	Q1. Title	Y	Y	Y	Y	Y	Y	Y	Y	Y	Y	Y	Y	Y	Y	100
Abstract	Q2. Structured summary	Y	Y	Y	Y	Y	Y	Y	Y	Y	Y	Y	Y	Y	Y	100
Introduction	Q3. Rationale	Y	Y	Y	Y	Y	Y	Y	Y	Y	Y	Y	Y	Y	Y	100
	Q4. Objectives	Y	Y	Y	Y	Y	Y	Y	Y	Y	Y	Y	Y	Y	Y	100
Methods	Q5.Protocol and registration	N	N	N	N	N	N	N	N	N	N	N	Y	N	N	7.14
	Q6. Eligibility criteria	Y	Y	Y	Y	Y	Y	Y	Y	Y	Y	Y	Y	Y	Y	100
	Q7. Information sources	Y	Y	Y	Y	Y	Y	Y	Y	Y	Y	Y	Y	Y	Y	100
	Q8. Search	PY	Y	PY	PY	PY	PY	PY	PY	PY	PY	Y	PY	Y	PY	21.4
	Q9. Study selection	N	Y	Y	Y	N	Y	N	Y	Y	Y	N	Y	Y	N	64.3
	Q10. Data collection process	Y	Y	Y	Y	Y	Y	Y	Y	Y	Y	Y	Y	Y	Y	100
	Q11. Data items	Y	Y	Y	Y	Y	Y	Y	Y	Y	Y	Y	Y	Y	Y	100
	Q12. Risk of bias in individual studies	Y	Y	Y	Y	Y	Y	Y	Y	Y	Y	Y	Y	Y	Y	100
	Q13. Summary measures	Y	Y	Y	Y	Y	Y	Y	Y	Y	Y	Y	Y	Y	Y	100
	Q14. Synthesis of results	Y	Y	Y	Y	Y	Y	Y	Y	Y	Y	Y	Y	Y	Y	100
	Q15. Risk of bias across studies	Y	Y	Y	Y	Y	Y	Y	Y	Y	Y	Y	Y	Y	N	92.9
	Q16. Additional analyses	Y	Y	N	Y	Y	N	Y	Y	Y	Y	Y	Y	N	N	71.4
Results	Q17. Study selection	Y	Y	Y	Y	Y	Y	Y	Y	Y	Y	Y	Y	Y	Y	100
	Q18. Study characteristics	Y	Y	Y	Y	Y	Y	Y	Y	Y	Y	Y	Y	Y	Y	100
	Q19. Risk of bias within studies	Y	Y	Y	Y	Y	Y	Y	Y	Y	Y	Y	Y	Y	N	92.9
	Q20. Results of individual studies	Y	Y	Y	Y	Y	Y	Y	Y	Y	Y	Y	Y	Y	Y	100
	Q21. Synthesis of results	Y	Y	Y	Y	Y	Y	Y	Y	Y	Y	Y	Y	Y	Y	100
	Q22. Risk of bias across studies	Y	Y	Y	Y	Y	Y	Y	Y	Y	Y	Y	Y	Y	N	92.9
	Q23. Additional analysis	Y	Y	N	Y	Y	N	Y	Y	Y	Y	Y	Y	N	N	71.4
Discussion	Q24. Summary of evidence	Y	Y	Y	Y	Y	Y	Y	Y	Y	Y	Y	Y	Y	Y	100
	Q25.Limitations	Y	Y	Y	N	Y	Y	Y	N	Y	Y	N	Y	Y	N	71.4
	Q26. Conclusions	Y	Y	Y	Y	Y	Y	Y	Y	Y	Y	Y	Y	Y	Y	100
Funding	Q27. Funding	N	Y	N	N	N	Y	Y	N	Y	Y	Y	Y	Y	Y	64.3

Y, yes; PY, partial yes; N, no.

### Evidence quality

The GRADE system was used to evaluate 54 items related to outcomes in 14 SRs/MAs. The review found that overall, the quality of the evidence from most studies was not satisfactory. No high-quality evidence was found; 17 moderate-quality pieces of evidence, 25 low-quality pieces of evidence, and 12 very low-quality pieces of evidence. Risk of bias is the most common downgrading factor across all projects, which may be related to the acupuncturist's difficulty in turning a blind eye to the treatment assignments, followed by publication bias, imprecision, and inconsistency, with no items downgraded for Indirectness. More details are presented in Table 5.

**Table 5:** Results of GRADE assessment.

**Author(s), year**	**Outcomes**	**Studies (participants)**	**Limitations**	**Inconsistency**	**Indirectness**	**Imprecision**	**Publication bias**	**Quality**
Gong et al. (16)	Response							
	AT vs. MT	1(60)	−1	−1	0	0	−1	VL
	AT vs. WAT	1(62)	−1	−1	0	0	−1	VL
	AT vs. EAT	1(128)	−1	−1	0	0	0	L
	BAT vs. AAT	1(147)	−1	−1	0	−1	0	VL
	AT+MT vs. MT	2(140)	−1	−1	0	−1	0	VL
	AT vs. EAT+MT	1(68)	−1	−1	0	0	−1	VL
Zhang et al. (17)	Response							
	AT vs. MT	10(633)	−1	0	0	−1	0	L
	THI							
	AT vs. MT	3(173)	−1	0	0	−1	0	L
	AT vs. SAT	2(90)	−1	−1	0	−1	0	VL
Song et al. (18)	Response							
	AT vs. MT	9(1174)	−1	0	0	0	0	M
Ji et al. (19)	Response							
	AT vs. MT	7(678)	−1	0	0	0	−1	L
	AT+MT vs. MT	4(511)	−1	0	0	0	−1	L
	EAT+AI vs. MT	2(132)	−1	0	0	0	−1	L
	MT+AP vs. MT	2(196)	−1	0	0	0	−1	L
Meng et al. (20)	Response							
	AT vs. MT	8(851)	−1	0	0	0	0	M
	OAT vs. CAT	10(774)	−1	0	0	0	0	M
	AT+MT vs. MT	4(324)	−1	0	0	0	0	M
	AT+MT vs. AT	4(184)	−1	0	0	0	0	M
Nie et al. (21)	Response							
	AT vs. MT	3(250)	−1	0	0	−1	−1	VL
	AT+MT vs. MT	2(200)	−1	0	0	−1	−1	VL
	AT+CPM vs. MT	1(73)	−1	0	0	−1	−1	VL
Ma et al. (22)	Response							
	AT vs. MT	7(496)	−1	0	0	0	−1	L
	AT+CPM vs. MT	4(324)	−1	0	0	0	−1	L
	AT vs. SAT	4(244)	0	0	0	0	−1	M
Fang et al. (23)	Response							
	EAT vs. AT	6(443)	−1	0	0	0	−1	L
	EAT+CPM vs. MT	8(759)	−1	0	0	0	−1	L
	EAT vs. CT	14(1202)	−1	0	0	0	−1	L
Xie et al. (24)	Response							
	AT vs. MT	5(358)	−1	0	0	0	0	M
	AT vs. CM	2(122)	−1	0	0	0	0	M
	AT+MT vs. MT	2(155)	−1	0	0	0	0	M
	AT+CM vs. CM	3(334)	−1	0	0	0	0	M
Liu et al. (25)	Response							
	AT vs. SAT	2(66)	0	0	0	0	−1	M
	AT vs. MT	3(190)	0	0	0	−1	−1	L
	AT+CT vs. CT	1(64)	0	0	0	−1	−1	L
	EAT vs. MT	3(240)	0	0	0	−1	−1	L
	AT+MT vs. AT	1(54)	0	0	0	−1	−1	L
	AT+MT vs. MT	1(100)	0	0	0	0	−1	M
He et al. (26)	Response							
	EAT vs. AT	1(128)	−1	0	0	0	−1	L
	EAT vs. PAT	2(64)	0	0	0	0	−1	M
	EAT+CPM+PT vs. CPM+PT	1(60)	−1	0	0	0	−1	L
	EAT+CPM vs. MT	1(60)	−1	0	0	0	−1	M
Huang et al. (27)	VAS							
	AT vs. SAT	4(263)	0	0	0	−1	0	M
	AT vs. MT	1(64)	−1	0	0	−1	0	L
	WAT vs. MT	2(117)	−1	0	0	−1	0	L
	THI							
	AT vs. SAT	1(57)	0	0	0	−1	0	M
	WAT vs. MT	2(117)	−1	0	0	−1	0	L
	AT vs. MT	1(64)	−1	0	0	−1	0	L
	AT vs. CT	1(70)	−1	0	0	−1	0	L
	TSI							
	AT vs. SAT	2(142)	−1	0	0	−1	0	L
Kim et al. (28)	Response							
	AT vs. SAT	7(293)	0	0	0	−1	0	M
	AT vs. MT	2(150)	−1	0	0	−1	0	L
Park et al. (29)	Response							
	AT vs. SAT	2(75)	−1	−1	0	0	−1	VL
	VAS							
	AT vs. SAT	3(88)	−1	−1	0	0	−1	VL
	TA vs. CT	1(22)	−1	−1	0	0	−1	VL

VS, versus; AT, acupuncture; MT, medication therapy; WAT, warm acupuncture; EAT, electroacupuncture; BAT, body acupuncture; AAT, abdominal acupuncture; AI, acupoint injection; AP, acupoint application; CPM, Chinese patent medicine; SAT, sham acupuncture; OAT, other acupuncture; CAT, conventional acupuncture; CT, conventional therapy; PAT, placebo acupuncture; PT, Psychotherapy; VAS, visual analog scale; THI, tinnitus handicap inventory; TIS, tinnitus severity index; VL, very low; L, low; M, moderate.

### Overlap between included reviews

**Figure 2:**
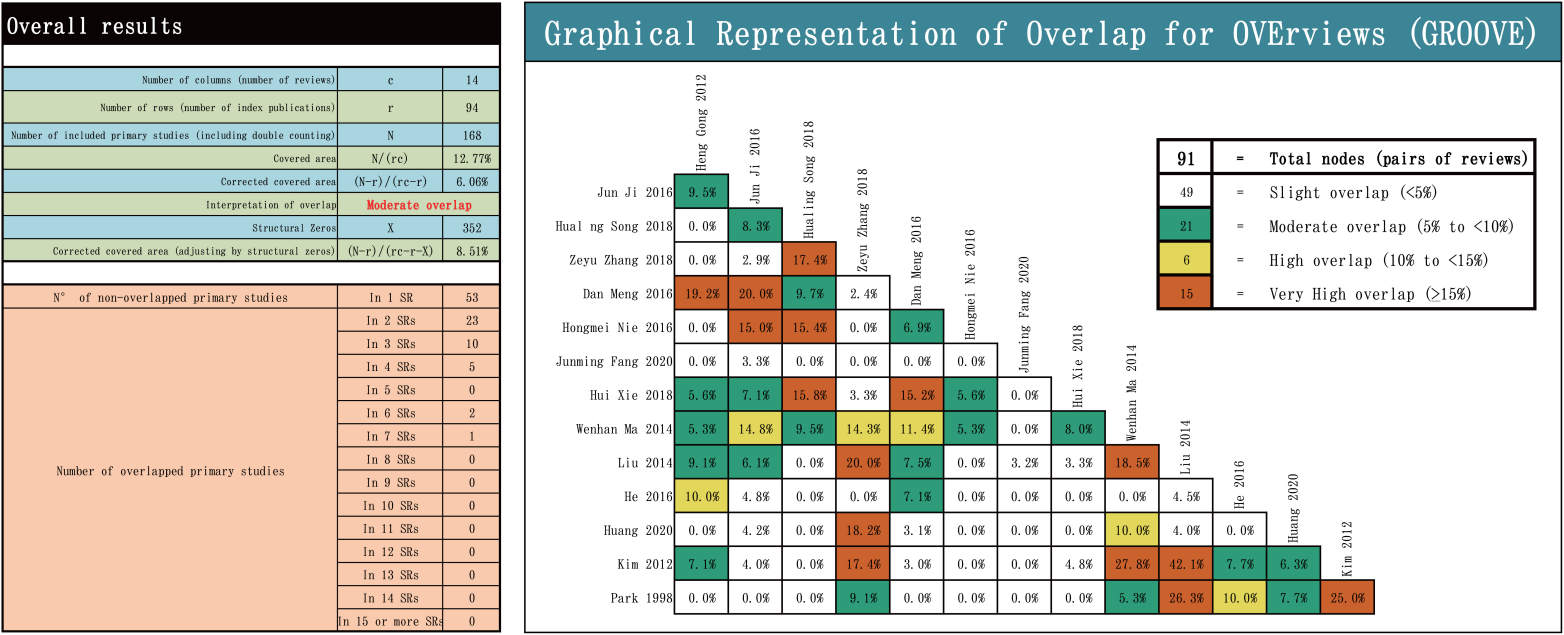
Overlapping of the included reviews.

Graphical Representation of Overlap for OVErviews not only computes the overall CCA but also provides a new graphical representation of the overlap between each pair of possible SRs/MAs. We observed moderate overlap between the included reviews. There were 91 total nodes between reviews, of which 49 were slight overlaps, 21 were moderate overlaps, 6 were high overlaps, and 15 were very high overlaps. More details are presented in Figure 2.

### Efficacy evaluation

Eleven studies (16–25, 27) reported positive results, and three studies (26, 28, 29) reported negative results, with positive results indicating that the acupuncture group was superior to the control group and negative results indicating that no significant difference between the acupuncture group and the control group.

All positive results reported the effectiveness of acupuncture for tinnitus, with nine studies (16–23, 25) comparing the effects of acupuncture with other treatments and two studies (24, 25) comparing the effects of electroacupuncture with other treatments. Nine studies (16–23, 25) compared the effectiveness of acupuncture and medication for tinnitus and showed that acupuncture was superior to medication. Four studies (16, 19, 20, 22) compared the efficacy of acupuncture plus medication with alone or acupuncture alone, showing that acupuncture plus medication was superior to medication or acupuncture. However, another study (21) showed that the combined effect of acupuncture plus medication was not significantly different from the overall efficacy. No conclusion was reached that acupuncture plus medication was better than medication. One study (25) reported that an analysis of pooled data seemed to suggest that acupuncture had some superiority over conventional therapy, as there was a significant difference in outcomes between the two groups. However, in this study (25), no significant differences were found when comparing the results of manual or electroacupuncture with conventional therapy and manual and electroacupuncture with medication. In another study (23), electroacupuncture was found to be more effective than traditional acupuncture. One study (27) reported that acupuncture had no significant effect on the primary outcome of VAS scores compared to controls; however, acupuncture had a relatively positive effect on the secondary outcomes of THI and TSI scores, which were significantly different from controls.

All negative (26, 28, 29) results reported no significant advantage of acupuncture compared to controls. Two studies (28, 29) reported that acupuncture showed no significant improvement in the efficacy of tinnitus compared to sham and placebo acupuncture. Another study showed no convincing evidence that electroacupuncture was beneficial for the treatment of tinnitus due to the small sample size and low methodological quality.

## Discussion

### Summary of evidence

In recent years, the number of SRs/MAs for acupuncture treatment of tinnitus has been increasing, due to differences in evaluation systems, resulting in inconsistent quality and unsatisfactory results. To further summarize the quality of evidence, reporting, and methodological quality of SRs/MAs for acupuncture for tinnitus, we conducted a detailed and comprehensive summary of the 14 studies.

It was found that among the 14 included SRs/MAs, 11 (78.6%) studies reported that acupuncture was effective in the treatment of tinnitus, and 3 (21.4%) studies reported that no firm conclusions were reached on the effectiveness of acupuncture in the treatment of tinnitus. All of the studies published in Chinese showed that acupuncture treatment for tinnitus was positive and effective, while three-fifths of the English papers showed that the efficacy of acupuncture treatment was inconclusive. The majority of SRs/MAs published in Chinese were mainly included in clinical trials published in Chinese, and only three SRs/MAs published in Chinese included a small number of clinical trials published in English, which may be the reason why all SRs/MAs published in Chinese had positive results. The GROOVE tool was used to extract and analyze all original RCTs included in SRs/MAs for acupuncture for tinnitus. Overall, the overlap between SRs/MAs that we included was moderate. According to the overlapping distribution area in Figure 2, most of the overlaps > 5% occurred between studies published in the same language. This finding suggests that studies published in the same language may have similar results, which could explain the difference in results between studies published in English and Chinese.

Vickers et al. (30) found that in some specific countries and regions (such as China, Japan, Korea, and Russia/Soviet Union), the results of published papers in the field of acupuncture may show a trend of positive results. It is well known that acupuncture originated in China 3,000 years ago, and was gradually introduced to Japan, Korea, and Southeast Asian countries; it was not until the 18th century that acupuncture research emerged in Europe and the United States (31). From a cultural and historical perspective, East Asian countries are more familiar with alternative and complementary medicine (32). Therefore, as the results of the overview found, the selection of acupoints, courses of treatment, manipulation, and acupuncturist proficiency in clinical trials published in Chinese may be more appropriate. However, this result must be treated with caution due to the low quality of the included studies, many of which suffer from methodological shortcomings, low quality of evidence, and the risk of bias. It is worth noting that although nearly 80% of the studies showed the efficacy of acupuncture in the treatment of tinnitus, the authors of the SRs/MAs seemed reluctant to draw definitive conclusions on this result because of the small sample sizes and methodological shortcomings of the included clinical trials. Meanwhile, the results of this review show that the methodological quality and evidence quality of almost all reviews are unsatisfactory, and based on the current evidence we, cannot draw firm conclusions about the efficacy of acupuncture in the treatment of tinnitus. There is a need for more rigorous, standardized, and comprehensive SRs/MAs to provide strong evidence for a convincing conclusion.

Currently, no medication has been approved by the Food and Drug Administration (FDA) or the European Medicines Agency (EMA) for the treatment of tinnitus (33). Although there is no cure for primary tinnitus, a wide range of treatments have been used and studied in an attempt to provide relief from symptoms. Unfortunately, although there are many treatments, evidence-based medicine still does not provide the most authoritative evidence for the treatment of tinnitus (12). Based on psychological intervention measures, especially those based on cognitive behavior therapy (CBT) intervention measures are often considered to be the most effective treatment of tinnitus; however, this approach is designed to reduce the pain associated with tinnitus, not to reduce tinnitus itself (34). In clinical practice, many patients may be reluctant to choose CBT or reject it outright due to practical considerations, and acupuncture is the only treatment that has clinical evidence of the improved quality of life in patients with tinnitus (28). The mechanism of acupuncture in the treatment of tinnitus is complex, and it has been reported that acupuncture has been evaluated for the possibility of affecting cochlear blood flow, which has been considered one of the causes of tinnitus (8). However, in many clinical trials, acupuncture points are not around the ear, but far away from the ear, such as the extremities or chest and back, which has a limited impact on cochlear blood flow. Therefore, in the process of clinical research, the selection of a rigorous and reliable randomization method, standard and sufficient sample size, and appropriate control group are the necessary conditions for clinical trials of acupuncture treatment of tinnitus, among which the careful selection of appropriate acupuncture placebo control group is one of the most important links. Acupuncture as a manual physical therapy method inevitably involves contact with patients, which requires balancing the background factors of acupuncture and control groups with the details of sham acupuncture in control groups to better measure the power of our treatment regimen and increase the internal and external validity of the findings (35).

## Limitations

To the best of our knowledge, this is the first overview of a systematic review of acupuncture for tinnitus and the first to provide a comprehensive evidence base for the clinical practice of acupuncture for tinnitus using the AMSTER-2 and GRADE assessment tools. However, we should acknowledge some objective limitations. First, evaluating the methodological quality and quality of evidence is subjective. Even if we evaluated each item of the evaluation system in detail and objectively, guidelines or authoritative third parties judged the disputes. The results may still be partially different. Second, we only included studies published in full text in both Chinese and English, which is likely to have a certain degree of risk of bias. Third, we did not conduct advance review registration on the PROSPERO website, which may have led to the risk of bias. Finally, due to the disease characteristics of tinnitus, the primary outcome measure of the RCTs included in this study was subjective clinical evaluation, which may have an impact on treatment outcomes.

## Conclusion

In conclusion, acupuncture seems to be a positive and effective treatment for tinnitus. However, the methodological quality and evidence for SR/MA in the included studies were generally low, and this result must be considered cautiously. Therefore, more high-quality, large-scale, multi-center randomized controlled trials are needed in the future to verify the effectiveness of acupuncture in the treatment of tinnitus.

## Data availability statement

The original contributions presented in the study are included in the article/Supplementary material, further inquiries can be directed to the corresponding author.

## Author contributions

XX and ZL planned, designed the study, assessed the quality, and summarized the evidence. XX wrote the original draft. HX reviewed and edited the manuscript. YZ and TG screened potential studies and extracted data from the included studies. All authors contributed to the article and approved the submitted version.

## Conflict of interest

The authors declare that the research was conducted in the absence of any commercial or financial relationships that could be construed as a potential conflict of interest.

## Publisher's note

All claims expressed in this article are solely those of the authors and do not necessarily represent those of their affiliated organizations, or those of the publisher, the editors and the reviewers. Any product that may be evaluated in this article, or claim that may be made by its manufacturer, is not guaranteed or endorsed by the publisher.
