# Supplementary material for: Effects of acupuncture on the outcome of tinnitus: An overview of systematic reviews
Source: Front Neurol. 2022 Nov 18;13:1061431. doi: 10.3389/fneur.2022.1061431 (PMC9716106; doi:10.3389/fneur.2022.1061431)
Supplement: Supplementary file 1 [file Table_1.DOCX]

**Appendix 1. Search strategies of each database**

The following database will be searched from inception to March 2022.

**Database 1 China National Knowledge Infrastructure (CNKI)**

SU=('耳鸣' +'主观性耳鸣'+'特发性耳鸣'+'慢性耳鸣'+'急性耳鸣'+'躯体性耳鸣'+'搏动性耳鸣')) AND SU=('针刺'+'针灸'+'耳针'+'体针'+'舌针'+'电针'+'温针灸'+'腹针'+'头针') AND SU=('荟萃分析'+'系统综述'+'系统评价'+'Meta分析')

**Database 2 Wanfang Database**

(耳鸣 or 主观性耳鸣 or 特发性耳鸣 or 慢性耳鸣 or 急性耳鸣 or 躯体性耳鸣 or 搏动性耳鸣) and (针刺 or 针灸or耳针or体针or舌针or电针or温针灸or腹针or头针) and (荟萃分析 or 系统综述 or 系统评价 or Meta分析)

**Database 3 Chongqing VIP**

(U=耳鸣 OR U=主观性耳鸣 OR U=特发性耳鸣 OR U=慢性耳鸣 OR U=急性耳鸣 OR U=躯体性耳鸣 OR U=搏动性耳鸣) AND (U=针刺 OR U=针灸 OR U=耳针 OR U=体针 OR U=舌针 OR U=电针 OR U=温针灸 OR U=腹针 OR U=头针) AND (U=荟萃分析 OR U=系统综述 OR U=系统评价 OR U=Meta分析)

**Database 5 Sino-Med**

1 "耳鸣" [不加权:扩展]

2 "主观性耳鸣"[常用字段:智能] OR "特发性耳鸣"[常用字段:智能] OR "慢性耳鸣"[常用字段:智能] OR "急性耳鸣"[常用字段:智能] OR "躯体性耳鸣"[常用字段:智能] OR "搏动性耳鸣"[常用字段:智能]

3 1 OR 2

4 "针刺疗法" OR "针刺" OR "针灸疗法" [不加权:扩展]

5 "针刺"[常用字段:智能] OR "针灸"[常用字段:智能] OR "耳针"[常用字段:智能] OR "电针"[常用字段:智能] OR "体针"[常用字段:智能] OR "舌针"[常用字段:智能] OR "温针灸"[常用字段:智能] OR "头针"[常用字段:智能] OR "腹针"[常用字段:智能]

6 4 OR 5

7 "Meta分析"[不加权:扩展]

8 "Meta分析"[常用字段:智能] OR "系统评价"[常用字段:智能] OR "荟萃分析"[常用字段:智能] OR "系统综述"[常用字段:智能]

9 7 OR 8

10 3 AND 6 AND 9

**Database 6 Pubmed**

1 Tinnitus [Mesh]

2 Tinnitus[Title/Abstract] OR Tinnit*[Title/Abstract] OR Ear and (Ring* or Buzz* or Roar* or Click* or Puls*)[Title/Abstract] OR Somatic Tinnitus [Title/Abstract] OR Idiopathic Tinnitus [Title/Abstract] OR Chronic Tinnitus [Title/Abstract] OR Acute Tinnitus [Title/Abstract] OR Pulsatile Tinnitus[Title/Abstract] OR Subjective Tinnitus [Title/Abstract]

3 1 OR 2

4 Acupuncture[Mesh]

5 Acupuncture[title/abstract] OR Dry Needling[title/abstract] OR Acupotomy[title/abstract] OR Acupotomies[title/abstract] OR Electroacupuncture[title/abstract] OR Body Acupuncture[title/abstract] OR Ear Acupuncture [title/abstract] OR Scalp Acupuncture [title/abstract] OR Warming Needle [title/abstract]

6 4 OR 5

7 Meta-Analysis as Topic[Mesh]

8 Systematic review[Title/Abstract] OR Meta-Analysis[Title/Abstract] OR meta-analyses[Title/Abstract] OR metaanalysis[Title/Abstract]

9 7 OR 8

13 3 AND 6 AND 9

Database 4 Web of science

Web of science：TS=(‘Tinnitus’ OR ‘Somatic Tinnitus’ OR ‘Idiopathic Tinnitus’ OR ‘Chronic Tinnitus’ OR ‘Tinnit*’ OR ‘Acute Tinnitus’ OR ‘Pulsatile Tinnitus’ OR ‘Subjective Tinnitus’) AND TS=(‘Acupuncture’ OR ‘Pharmacoacupuncture’ OR ‘Acupotomy’ OR ‘Acupotomies’ OR ‘Pharmacopuncture’ OR ‘needle’ OR ‘needling’ OR ‘dry-needling’ OR ‘body-acupuncture’ OR ‘electroacupuncture’ OR ‘electro-acupuncture’ OR ‘auricular acupuncture’ OR ‘warm needle) AND TS=(‘systematic review*’ OR ‘meta-analysis’ OR ‘meta-analyses’ OR ‘meta analysis’)

**Database 7 Cochrane Library**

1 Mesh descriptor: [Tinnitus]explode all trees;

2 Tinnitus:ti,ab,kw or Tinnit*:ti,ab,kw or Somatic Tinnitus:ti,ab,kw or Idiopathic Tinnitus:ti,ab,kw or Chronic Tinnitus:ti,ab,kw or Acute Tinnitus:ti,ab,kw or Pulsatile Tinnitus:ti,ab,kw or Pulsatile Tinnitusti,ab,kw (Word variations have been searched)

3 1 or 2

4 Mesh descriptor: [Acupuncture] explode all trees;

5 acupuncture:ti,ab,kw or acupotomy:ti,ab,kw or acupotomies:ti,ab,kw or needle:ti,ab,kw or needling:ti,ab,kw or "dry- needling:ti,ab,kw or body-acupuncture:ti,ab,kw or electroacupuncture:ti,ab,kw or electro-acupuncture:ti,ab,kw or auricular acupuncture:ti,ab,kw or warm needle:ti,ab,kw or pharmacoacupuncture:ti,ab,kw or pharmacopuncture:ti,ab,kw (Word variations have been searched);

6 7 or 8

9 Mesh descriptor: [Meta-Analysis as Topic] or [Meta-Analysis]explode all trees;

10 systematic review:ti,ab,kw or Meta-Analysis:ti,ab,kw or meta analysis:ti,ab,kw or meta-analyses:ti,ab,kw (Word variations have been searched);

11 9 or 10

12 3 and 6 and 9

**Database 8 EMBASE**

1 'Tinnitus'/exp

2 'Tinnitus':ab,ti OR 'Tinnit*':ab,ti OR ' Somatic Tinnitus':ab,ti OR 'Idiopathic Tinnitus':ab,ti OR ' Chronic Tinnitus':ab,ti OR 'Acute Tinnitus':ab,ti OR 'Pulsatile Tinnitus':ab,ti OR ' Pulsatile Tinnitus':ab,ti

3 1 OR 2

4 'acupuncture'/exp

5 'acupuncture':ab,ti OR 'acupotomy':ab,ti OR 'acupotomies':ab,ti OR 'needle':ab,ti OR 'needling':ab,ti OR 'dry-needling':ab,ti OR 'body-acupuncture':ab,ti OR 'electroacupuncture':ab,ti OR 'electro-acupuncture':ab,ti OR 'auricular acupuncture':ab,ti OR 'warm needle':ab,ti OR 'pharmacoacupuncture':ab,ti OR 'pharmacopuncture':ab,ti

6 4 OR 5

7 'meta analysis'/exp OR 'meta analysis (topic) '/exp OR 'systematic review'/exp OR 'systematic review (topic) '/exp

8 'systematic review':ab,ti OR 'Meta-Analysis':ab,ti OR 'meta analysis':ab,ti OR 'meta-analyses':ab,ti OR 'metaanalysis':ab,ti

9 7 or 8

10 3AND 6 AND 9
